# Fabrication of Stabilized Fe–Mn Binary Oxide Nanoparticles: Effective Adsorption of 17β-Estradiol and Influencing Factors

**DOI:** 10.3390/ijerph15102218

**Published:** 2018-10-11

**Authors:** Qimeng Ning, Zhihong Yin, Yunguo Liu, Xiaofei Tan, Guangming Zeng, Luhua Jiang, Shaobo Liu, Sirong Tian, Ni Liu, Xiaohua Wang

**Affiliations:** 1 College of Environmental Science and Engineering, Hunan University, Changsha 410082, China; ningqimeng@163.com (Q.N.); hnuliuyunguo@gmail.com (Y.L.); Tanxf@hnu.edu.cn (X.T.); zgming@hnu.edu.cn (G.Z.); Jiangluhua@hnu.edu.cn (L.J.); Tsirong@hnu.edu.cn (S.T.); meet_liuni@hnu.edu.cn (N.L.); Wangxiaohua6999@126.com (X.W.); 2 Key Laboratory of Environmental Biology and Pollution Control (Hunan University), Ministry of Education, Changsha 410082, China; 3 School of Architecture and Urban Planning, Hunan City University, Yiyang 413000, China; 4 School of Architecture and Art, Central South University, Changsha 410082, China; Liushaobo23@163.com; 5 School of Metallurgy and Environment, Central South University, Changsha 410083, China

**Keywords:** stabilized Fe–Mn binary oxide nanoparticles, oxidation, 17β-estradiol, adsorption

## Abstract

Fe–Mn binary oxide nanoparticles (FMBON) were reported to be high performance as adsorbent for pollutants removal from aqueous solution. However, there are still limitations in practice application due to the FMBON tend to aggregate into the micro millimeter level. In order to avoid the agglomeration of nanoparticles, this work synthesized the stabilized Fe–Mn binary oxide nanoparticles (CMC-FMBON) by using water-soluble carboxymethyl celluloses (CMC) as the stabilizer. The characteristics of CMC-FMBON and FMBON were measured by using Transmission electron microscopy (TEM), X-ray diffraction (XRD), Fourier transform infrared (FTIR) spectroscopy, and Zeta potential. This work systematically investigated the adsorption capacity of CMC-FMBON for 17β-estradiol (E2) and the influences of external environmental factors on E2 removal. The results indicated that CMC-FMBON had much smaller particles, wider dispersion and larger surface area than the FMBON. CMC-FMBON showed better adsorption performance for E2 than FMBON with the maximum adsorption capacity of CMC-FMBON and FMBON were 124.10 and 98.14 mg/g at 298 K, respectively. The experimental data can be well fitted by the model of pseudo-second-order and Langmuir model. The E2 removal by CMC-FMBON was obviously dependent on pH with the maximum adsorption occurring when the pH was acidic. The removal capacity of CMC-FMBON increased when enhancing ionic strength in solution. Background electrolytes promoted slightly E2 adsorption process whereas the presence of humic acid inhibited the E2 removal. π-π interactions, hydrogen bonds, and oxidation might be responsible for E2 removal. This research suggested that the CMC-FMBON has been considered to be a cost-efficient adsorbent for removing E2 from water.

## 1. Introduction

**Figure 1:**
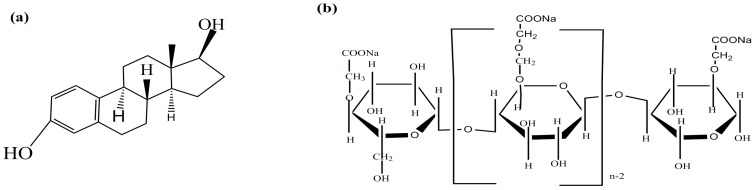
Conceptualized representation of the 17β-estradiol (E2) (**a**) and carboxymethyl celluloses (CMC) molecular structure (**b**).

Endocrine Disrupting Chemicals (EDCs) have caused great concern in recent years because of their presence not only in aquatic environments but also in industrial wastewater [1,2]. The use of 17β-estradiol (E2), as a type of EDCs, could pose potential risks to the natural ecosystem and humans [3]. It has been reported that E2 can cause various damage and cancer to the human health system by changing the function of the endocranium even at low concentrations [4]. The negative effects of E2 on organisms included inhibiting the production of fish eggs and causing sexual reversal of males [5]. Consequently, removing E2 from water has become an urgent environmental issue. Figure 1a presents the conceptualized representation of the molecular structure of E2.

Various physical and chemical methods have been used to remove E2, including photo-catalytic degradation [6], photo-fenton degradation [7], adsorption [8,9,10], and biodegradation [11]. Adsorption has been considered as a low cost and high efficiency technology for pollutant remediation [12,13,14,15,16,17] among these available methods. Previous investigations revealed that many different adsorption materials have been used to remove E2 such as granular activated carbon (GAC) [18], powdered activated carbons (PAC) [19], multi-walled carbon nanotubes [20], and graphene [12,13]. However, these sorbents have some problems of either high environmental impact or low adsorption efficiency, which hindered practical application in the pollutant’s removal.

In recent years, various manganese oxides/hydroxides for example Fe–Mn binary oxides nanoparticles (FMBON), manganese oxide (MnO_2_), and birnessite have been applied to the pollutants research. It was reported that FMBON was a highly efficient and low-pollution adsorbent for the treatment of pollutants due to its high adsorption capacity, low costs and environmental impacts [3,21,22,23]. Liu et al. [24] prepared FMBON and reported that the adsorbent had great interests for tetracycline removal from aqueous solution. A study conducted by Zhang et al. [25] showed that FMBON was a promising candidate for phosphate ions removal. However, there are still some limitations of applying FMBON in the pollutants elimination because of the FMBON tend to aggregate into the micro millimeter level or larger, which will result in the loss of chemical reactivity and soil fluidity of FMBON. Agglomeration can decrease interface free energy and specific surface area of adsorbent, resulting in reduced ability to remove contaminants. The phenomenon of metal nanoparticles aggregation was mainly by direct interparticle interaction, for example magnetic interactions and Van der Waals forces [26]. To avoid particle agglomeration, many materials were synthesized, such as trisodium citrate based magnetite nanocomposite (Fe_3_O_4_-TSC) [27], Fe_3_O_4_@TAS [28] and magnetic metal—organic framework nanocomposite (Fe_3_O_4_@AMCA-MIL53(Al)) [29]. Researchers have attempted other materials as a stabilizer to stabilized FMBON. Various stabilizers have been found to be applied in the remediation of pollutants such as thiols, carboxylic acids, starch, polymers, and surfactants. However, some of them are limited as stabilizers to prevent nanoparticles from agglomeration. For example, certain polymers may not work properly in aqueous solution, and some of they are cost-prohibitive and not environmentally friendly.

An ideal stabilizer should have the following conditions: (a) it can interact with the nanoparticles and thus inhibit their growth; (b) it has the advantages of being environmentally friendly and cost-effective; and (c) it has good soil mobility and chemical reactivity. Recently, carboxymethyl celluloses (CMC) have gained more and more attention because of their excellent stability as a stabilizer. CMC with macromolecular framework structures was commonly used in food processing and was a water-soluble organic substance. Figure 1b conceptually shows the molecular structure of CMC. CMC is a polyelectrolyte carrying carboxylate groups, capable of promoting the dispersion of the nanoparticles, and enhancing the adsorption capacity for pollutants in water [8]. CMC as an effective stabilizer has been successfully applied to the preparation of nanoparticles materials such as iron oxide, Ag, and Pb nanoparticles. However, research information on the synthesis of stabilized Fe–Mn binary oxides nanoparticles by using CMC as a stabilizer has been rarely reported. In addition, to our best knowledge, there were few investigates about stabilized Fe–Mn binary oxides nanoparticles for E2 removal in water. Moreover, the adsorption process of E2 can be influenced by external environmental factors such as the content of CMC, reactive temperature, the initial pH, coexisted ions, ionic concentration, and humic acid. However, there are few systematic studies on the effects of external environmental factors on E2 adsorption.

In this work, we synthesized FMBON and stabilized Fe–Mn binary oxides (CMC-FMBON) by using CMC as a stabilizer for E2 removal in aqueous solution. The specific goals of this work were to: (1) synthesize a new class of CMC-FMBON using CMC as stabilizer; (2) measure characteristics of CMC-FMBON and FMBON by using Transmission electron microscopy (TEM), Fourier transform infrared (FTIR) spectroscopy, X-ray diffraction (XRD), and Zeta potential, respectively; (3) systematic study the adsorption capacity of CMC-FMBON for E2 and the effect of stabilizer content on E2 removal process; (4) explore external environmental factors on E2 removal process including initial pH, ionic strength, coexisting ions, and humic acid; and (5) evaluate the potential mechanism of E2 removal onto CMC-FMBNO.

## 2. Materials and Methods

### 2.1. Materials

The E2 (C_18_H_24_O_2_, molecular weight 228.29, 98% in purity) was purchased from Sigma–Aldrich Corp, (St. Louis, MO, USA). CMC (sodium salt, M_W_ = 90,000) was purchased from Shan Pu Chemical Corp, (Shanghai, China). The other chemicals were obtained from Municipality Kemi’ou Chemical Reagent Corp (Tianjin, China). All other chemicals were analytical grade or higher. All solutions were prepared by using deionized water (18.2 MΩ cm). 

### 2.2. Synthesis of CMC-FMBON

The material were prepared by the modified method [30] and the process were descried in the following: the stocks solutions were firstly prepared: 5.95 mM KMnO_4_, 1.12 mM FeCl_2_, and 1 wt. % CMC. Different volumes (0–20 mL) of CMC solution were added to the FeCl_2_ solution (160 mL) and mixed for 20 min. Then, KMnO_4_ solution (10 mL) was added dropwise into the mixtures of FeCl_2_ and CMC. The redox reaction was completed under magnetic stirring:

(1) $${3\mathrm{Fe}}^{2+}{+\mathrm{Mn}}^{7+}\to {3\mathrm{Fe}}^{3+}{+\mathrm{Mn}}^{4+}\;$$

The pH of the mixture was maintained at 7.5 through 0.1 M NaOH and HCl, and the total mixture volume was kept at 200 mL by adding ultrapure water. The nanoparticle suspensions contained 0.017 g/L Mn, 0.05 g/L Fe and 0–0.1% CMC. The CMC-FMBON were allowed to grow for 2 h at 298 K and finally stored in refrigerator for subsequent analysis. We also prepared FMBON without CMC for comparison.

### 2.3. Characterization of CMC-FMBON

The materials were characterized by TEM (TEM, Tecnai G2 F20, FEI, Hillsboro, OR, USA) running under the 200 kV [31]. The point of zero charge of materials were measured by Zetasizer (Nano-ZS90, Malvern, UK) [32]. The samples were prepared with ultrasonification 5 mL CMC-FMBON and deionized water (20 mL). The pH of the solution was adjusted to 2–10 by negligible volumes of 0.1 M NaOH and HCl. The structural characteristics of CMC-FMBON was measured on an X-ray diffractometer (XRD, D/max-2500, Rigaku, Tokyo, Japan). The functional groups of samples were characterized using Fourier transforms infrared (FTIR) spectrophotometer (Nicolet 6700 spectrometer, Thermo Fisher, Waltham, MA, USA).

### 2.4. E2 Removal from Aqueous Solution: Batch Adsorption Tests

A series of adsorption experiments were carried to test the removal capacity of E2 in aqueous solution. The E2 stock solution was prepared by dissolving E2 powder into a methanol solution. In addition, the methanol concentration was limited below 0.1% to avoid the co-solvent influence. The desired concentration of E2 was prepared by further diluting the stock E2 solution with ultrapure water. The E2 and CMC-FMBON mixed suspensions were shaken on a shaker at 160 rpm for 600 min. After the mixture was centrifugal separated at 8000 rpm for 20 min, thus the suspension was separated by a 0.45 μm membrane [31]. The adsorption capacity of CMC-FMBON for E2 was calculated by conducting mass balance between the E2 concentrations in the initial and final solutions. The E2 concentration was measured by An F-4500 fluorescence spectrophotometer (Hitachi, Tokyo, Japan) [33]. The measure methods of E2 was described in the Supplementary Material. Adsorption capacity was calculated by calculating the concentration of initial and equilibrium. The adsorption capacity of E2 by adsorbents were calculated as follows:

(2) $$q_{e}=\frac{\left(C_{o}-C_{e}\right)\times V}{m}\;$$

where *C*_o_ and *C*_e_ (mg/L) are the initial and equilibrium concentration of E2, respectively; *m* is the weight of sorption (g); *V* (mL) is the volume of E2.

To studied the influences of the CMC content, the materials of CMC-FMBON were prepared with various content of CMC (0, 0.025, 0.05, 0.075, and 0.1 wt. %). The experiments were carried out by following initial conditions: initial E2 concentration = 6 mg/L, contact time = 600 min and T = 298 K. Batch kinetic experiments were constructed to test the adsorption capacity for removing E2 from aqueous solution under different time periods. The following initial condition were applied in the batch kinetic tests: E2 concentration = 6 mg/L, contact time = 600 min, T = 298 K and CMC concentration = 0.075%. Similarly, E2 sorption isotherm were determined using equilibrium batch tests with three different reaction temperature (298, 318, and 338 K). The following experiment conditions were similar process of kinetics experiment except initial E2 concentration were changed (1, 2, 3, 4, 5, 6, 7 and 8 mg/L). Batch experiments used to measure the impact of solution pH on E2 (6 mg/L) removal process from aqueous system. The experiment was performed by varying the initial pH range from 2.0 to 10.0 in the solution. The pH was adjusted by adding negligible volumes of 0.1 M NaOH and HCl. To investigate the effect of ionic concentration, various concentrations of NaCl (0.02, 0.04, 0.06, 0.08, 0.10 M) were added to the E2 solution (6 mg/L). To studied the influence of coexisting ions (Na^+^, K^+^, Mg^2+^, Ca^2+^, Cl^−^, NO_3_^−^, SO_4_^2−^, and PO_4_^3−^), the ions solution were introduced to the E2 solution (6 mg/L) in the batch equilibrium experiments. To investigated the impact of humic acid on the adsorption of E2 by CMC-FMBON, the experiments was carried out through adding different concentration of humic acid (0–10 mg/L) to the E2 solution (6 mg/L) for each flask. All experiments were constructed in triplicate to ensure the accuracy of experiments results.

## 3. Results and Discussion

### 3.1. Characterization of FMBON and CMC-FMBON

**Figure 2:**
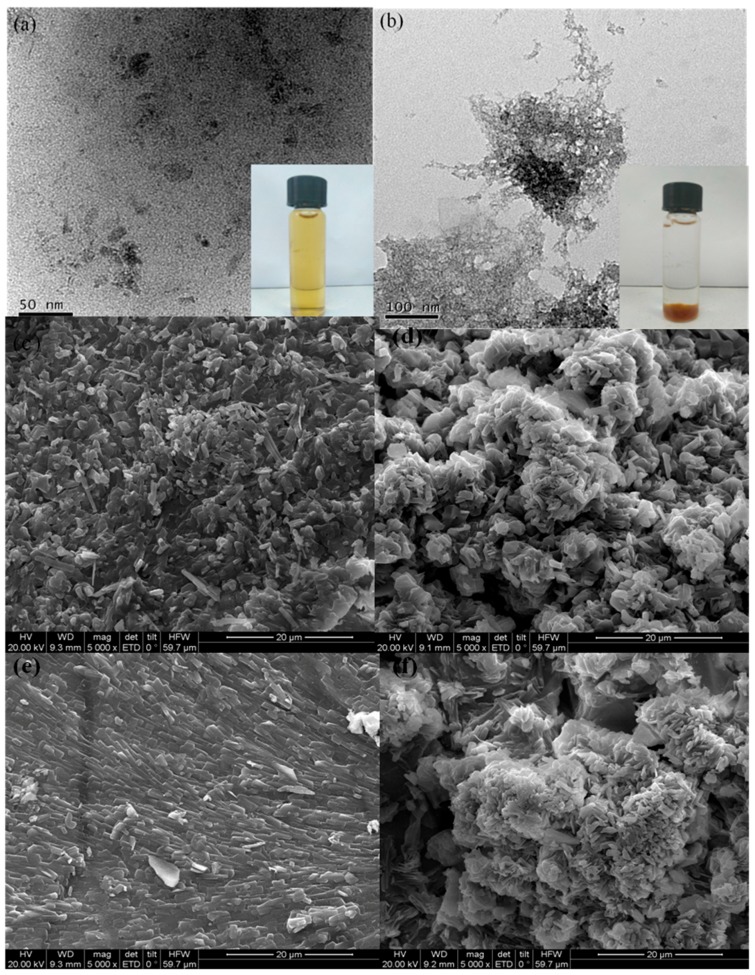
Transmission electron microscopy (TEM) images of (**a**) CMC-FMBON (carboxymethyl celluloses-Fe–Mn binary oxide nanoparticles) and (**b**) FMBON (Fe–Mn binary oxide nanoparticles), Scanning Electron Microscopy (SEM) images of FMBON (**c**), CMC-FMBON (**d**), after E2 adsorption FMBON (**e**) and CMC-FMBON (**f**).

The morphology studies of FMBON and CMC-FMBON were investigated by TEM and Scanning Electron Microscopy (SEM) as shown in Figure 2. The TEM images of FMBON and CMC-FMBON are displayed in Figure 2a,b. As seen in Figure 2a,b, the FMBON was covered with many aggregated small particles, which indicated the presence of a rough surface and porous structure. In contrast, CMC-FMBON had much smaller particles and wider dispersion, which indicated that the use of the CMC enabled the FMBON to be completely dispersible and easily transportable in aqueous solution. The SEM images of FMBON and CMC-FMBON before and after E2 adsorption are shown in Figure 2c–f. Compared to FMBON (Figure 2c), the CMC-FMBON (Figure 2d) is fully dispersed with better stabilization and much smaller particles. After E2 adsorption, the FMBON surface became flat (Figure 2e) and pore volume of CMC-FMBON decreased (Figure 2f), which indicated that E2 have been successfully adsorbed on the surface of adsorbents. The composition of FMBON and CMC-FMBON before and after E2 adsorption was quantified by EDX. The EDX spectra are shown in Figure S1. The elements of Fe, Mn, C, and O were detected in FMBON and CMC-FMBON surfaces. In addition, the specific surface area of CMC-FMBON and FMBON were 285 m^2^/g and 184 m^2^/g, respectively, which was similar with previous results [25]. Both the smaller average size and the narrower CMC-FMBON distribution indicated that the CMC can better inhibited the growth of the FMBON and thus obtained a higher specific surface area. The mixing of the main redox reaction products (iron and manganese binary oxides) between Mn(VII) and Fe(II) not only increases the formation of more nanoparticles, but opens up more specific surface area [34]. After E2 adsorption, the specific surface area of FMBON and CMC-FMBON decreased, which can be caused by the pollutant occupied the adsorption site on the surface of the adsorbent, resulting in a decrease in specific surface area. The results of specific surface area was consistent with the results of SEM images of FMBON and CMC-FMBON after E2 adsorption. In addition, it has been observed that FMBON precipitated within a few days, while CMC-FMBON remained dispersed in water for one week. The results indicated that CMC as stabilizer exhibited effective stability against nanoparticles aggregation in water. CMC-FMBON offered smaller particle size and more uniform particle size distribution, which was favorable to remove E2 in aqueous solution.

**Figure 3:**
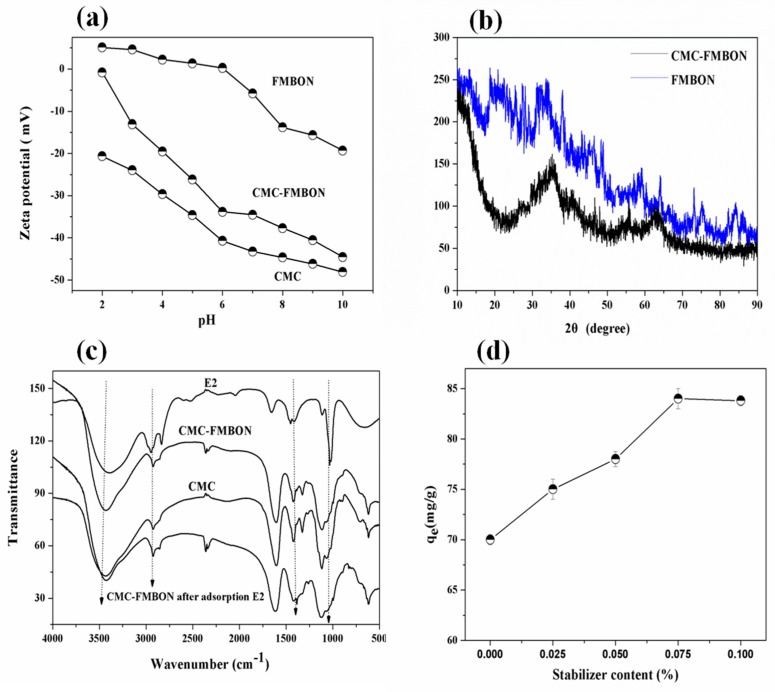
(**a**) Zeta potential as a function of pH for CMC (Carboxymethyl Celluloses), FMBON and CMC-FMBON. (**b**) X-ray diffraction (XRD) images of CMC-FMBON and FMBON. (**c**) Fourier transform infrared (FTIR) spectra of CMC and CMC-FMBON, E2 and CMC-FMBON before and after the reaction with E2. (**d**) Effect of stabilizer content on E2 removal by CMC-FMBON.

The zeta potential could affect the stability of colloid, electrostatic force between ions and the adsorption behavior in solution. Figure 3a shows the zeta potential for CMC, CMC-FMBON, and FMBON, respectively. As seen, CMC macromolecules surface carries a high density of negative charges. Increasing pH from 2.0 to 10.0 could increase the zeta potential from −21 to −46 mV. For the FMBON, the zeta potential values varied from +1.12 mV to −19 mV when the pH ranged from 2.0 to 10.0. According to the results, the pH of the point of zero charge (pH_pzc_) of FMBON was 6.0. It was reported that the pH_pzc_ of β-MnO_2_ and δ-MnO_2_ were 4.2 [35] and 2.5 [36], respectively, which were smaller than that of FMBON. The comparison results indicated that the surface of the FMBON was mainly dominated by iron oxides. The zeta potential of CMC-FMBON ranged from −2 to −44 mV with solution pH ranged from 2.0 to 10.0. Compared to FMBON, the zeta potential of CMC-FMBON rendered a much more negative surface potential, which indicated that the existence of the CMC greatly affected the surface potential. This might be ascribed to the CMC coating which had built up a negatively charged shell. Aggregation of the nanoparticles was inhibited by the electrostatic repulsion or steric hindrance between the CMC-coated nanoparticles. It is a protective CMC layer for FMBON.

Figure 3b exhibited the XRD patterns for CMC-FMBON and FMBON. The results clearly showed there are no significant peaks (intensity) in XRD pattern, which suggested that the non-crystalline nature structure for CMC-FMBON and FMBON. According to the results, the d-spacing was 2.60 Å and 1.48 Å. It showed that there are two broad peaks of ordered two-line ferrihydrite pattern at 35.0° and 64.5° [37], respectively, which indicated that CMC-FMBON exist mainly in amorphous form. These results were consistent with research by Yan et al. [38].

FTIR spectra of CMC-FMBON, FMBON, and CMC are shown in Figure 3c, respectively. Two peaks at 3400 and 3428 cm^−1^ were found for CMC-FMBON and CMC, respectively. It was worth noting that the –OH stretching bond shifted from 3428 to 3400 cm^−1^, which indicated an increase in strength of intermolecular hydrogen bonds [39]. Hydrogen bonds is vital for the binding of CMC to FMBON because of the abundance of –OH groups on CMC group. Peak at 1118 cm^−1^ could be assigned to the bending vibration of hydroxyl groups of iron hydroxides (Fe–OH) vibration for CMC-FMBON and FMBON [40]. The peak of 2925 cm^−1^ was ascribed to the stretching vibration of –CH_2_ bands [41]. The peaks around 1418 and 1620 cm^−1^ were assigned to asymmetric and symmetric vibrations of –COOH bands [42]. The bonding mechanism can be identified by asymmetric and symmetric stretches [Δ*v* = Δ(asym) − Δ(sym)] of the carboxylate group. If the value of Δ*v* < 110 cm^−1^, which suggested that the bonding mechanism is govern by bidentate chelation interaction; If the value of 140 < Δ*v* < 190 cm^−1^, which indicated that the bonding interaction is controlled by bidentate bridging; If the value of 200 < Δ*v* < 320 cm^−1^, the binding is controlled by monodentate interaction. In this work, Δ*v* was calculated to be 202 cm^−1^ (1620–1418 cm^−1^). Therefore, the binding of CMC particles and FMBON nanoparticles was primarily bound by monodentate interaction. The similar binding mechanism was reported by Feng He et al. [26]. Two peaks around 1600 cm^−1^ for CMC-FMBON and 1611 cm^−1^ for CMC were shown, respectively. This shift from 1611 to 1600 cm^−1^ could be assigned to decrease of covalent bonds due to the inhibition of the conjugation of –COOH when CMC attached to the FMBON surface. The FTIR results illustrated that the functional groups of CMC-FMBON were enhanced through adsorption of CMC onto the surface by hydroxyl and carboxyl groups. Besides, the FTIR spectra of CMC-FMBON after reaction as well as E2 are also showed in Figure 3c and the detailed description will be discussed in the following part.

### 3.2. The Removal Capacity of CMC-FMBON for E2

#### 3.2.1. Stabilizers Content

Figure 3d presented the adsorption ability of E2 as a function of type and content of CMC. The adsorption capacity steadily increased with the stabilizer concentration varied from 0 to 0.1%. The *q*_e_ (mg/g) values of CMC-FMBON change from 70.02 to 84.20 mg/g. According to the content of total Fe in the supernatants, the CMC-FMBON were stabilized when the CMC concentration was 0.04%, which was the minimum stabilizer content for complete nanoparticles stabilization, also referred to as the critical stabilization concentration (CSC). When the stabilizer contents were lower than CSC, the adsorption capacity increased steadily as the stabilizer content increased. This could be contributed to the fact that CMC could provide adsorption active sites for E2. However, the adsorption capacity decreases slightly while further increasing the stabilizer concentration. The reason could account for the results that the stabilized CMC-FMBON may have lost some of the reactive sites because the blocking effect of the stabilizer or sorption sites remaining saturated. Further increasing the content of CMC had litter effect on E2 adsorption, which indicated that the adsorption sites is increased because of smaller size and is balanced. This might prevent some adsorption sites and slow down the mass transfer [43]. The results was similar with Byungryul et al. studies [30].

#### 3.2.2. Adsorption Kinetics

**Figure 4:**
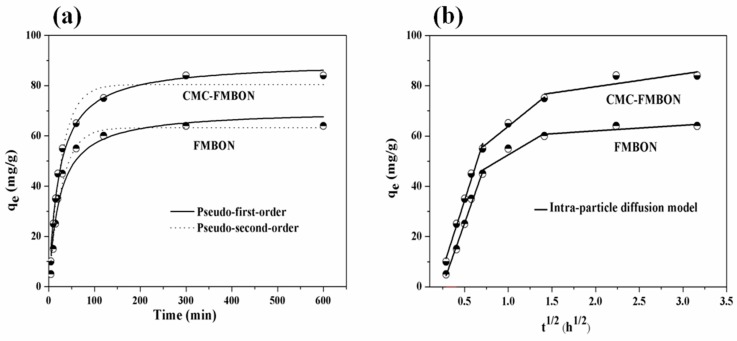
Kinetics of E2 removal using CMC-FMBON and FMBON, (**a**) Pseudo-first-order and pseudo-second-order model; (**b**) Intra-particle diffusion model.

The kinetic tests experiment were performed to determine the adsorption rate of E2 from aqueous solution by CMC-FMBON and FMBON. Figure 4a compared E2 adsorption rates of CMC-FMBON and FMBON. The equilibrium adsorption capacity of FMBON and CMC-FMBON were 60.32 and 84.20 mg/g, respectively. The high removal capacity of CMC-FMBON revealed the important role of CMC on adsorption process of E2. It could be found clearly that the adsorption amount rapidly increased in the first 100 min and then gradually decreased until it reached the equilibrium time at 600 min. The rapid adsorption rate at the beginning of adsorption was likely because of the availability of a lot of active sites on the adsorbent surface [44]. The fine particles of CMC-FMBON were conducive to the diffusion of E2 molecules from bulk solution onto the adsorbent surface. The similar phenomenon was also observed by removing selenite onto a stabilized Fe–Mn binary oxide [16]. Based on the results of adsorption kinetics, adsorption equilibrium time 600 min was used in the other batch experiments.

Furthermore, two modeling approaches (pseudo-first-order and pseudo-second-order model) were employed to interpret the E2 adsorption kinetics. These equations are given as follows:

Pseudo-first-order model:

(3) $$\ln(q_{e}-q_{t})=\ln q_{e}-k_{1}t\;$$

Pseudo-second-order model:

(4) $$\frac{t}{q_{t}}=\frac{1}{k_{2}q_{e}{}^{2}}+\frac{t}{q_{e}}\;$$

where *q*_e_ and *q*_t_ are the adsorbed capacity of E2 at equilibrium and different time periods, respectively. *k*_1_ (1/min) and *k*_2_ (g/mg min) are the sorption rate constants of the two model.

In Figure 4a, the kinetic curves of E2 onto CMC-FMBON and FMBON are presented, respectively. The kinetics parameters that were obtained from kinetics model are displayed in Table 1. It clearly showed the regression coefficients value of the pseudo-second-order model (*R*^2^ = 0.98) was higher than the pseudo-first-order model (*R*^2^ = 0.96) for E2 adsorption by CMC-FMBON. These results showed that the pseudo-second-order model was more suitable for the experimental data than the pseudo-first-order model. For FMBON, the pseudo-second-order model does describe the experiment results well due to its value of corresponding *R*^2^ is relatively higher. The result suggested that both E2 adsorbed onto the surface of CMC-FMBON and FMBON by chemical adsorption, and the chemical adsorption may be the rate-limiting mechanism.

To further investigate the diffusion mechanisms and potential rate controlling process, intra-particle diffusion model was fitted the experimental data. The equation of intra-particle diffusion model are shown as the following equation:

(5) $$q_{e}=k_{\mathrm{id}}t^{\frac{1}{2}}+C_{i}\;$$

where *k*_id_ is the rate constant of intra-particle diffusion (mg/g h^1/2^) and *C*_i_ is the intercept associated with the thickness of the boundary layer. Based on the intra-particle diffusion model, if intra-particle diffusion was involved in the adsorption process, then *q*_t_ versus t^1/2^ should be linear. 

The results of intra-particle diffusion model are displayed in Figure 4b, the plots are multi-linear including three sections: (a) film diffusion (mass transfer); (b) intra-particle diffusion; and (c) adsorption on the internal surface of the adsorbent. The first sharper slope of the curve was attributed to the diffusion of E2 from the bulk solution to the external adsorbent surface through film diffusion. The second section were corresponded to the intra-particle diffusion onto CMC-FMBON and FMBON. The third section exhibited a small slope, which indicated that the intra-particle diffusion begin to slow down. The phenomenon might be explained by low E2 concentration or less number of available adsorption sites on the CMC-FMBON and FMBON surfaces as the adsorption time increases. Therefore, the results indicated that both the intra-particle diffusion and film diffusion might involve in the adsorption process. The similar phenomenon were also observed in the process of adsorption of ciprofloxacin by similar Fe–Mn binary oxides nanoparticles [38].

#### 3.2.3. Adsorption Isotherm Experiment and Thermodynamic

**Figure 5:**
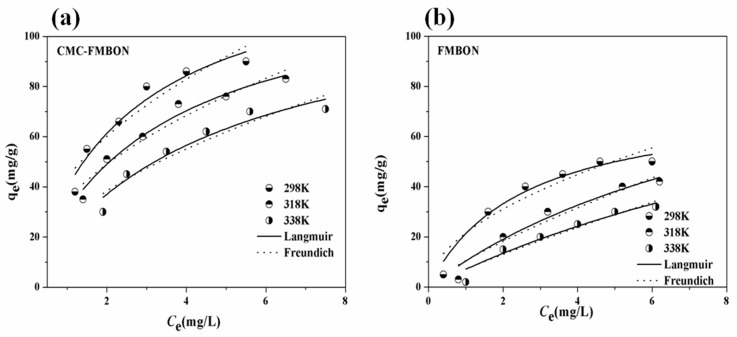
E2 sorption isotherms with (**a**) CMC-FMBON and (**b**) FMBON at three different temperature (298 K, 318 K, and 338 K).

Figure 5 presented the adsorption capacity of CMC-FMBON and FMBON for E2 adsorption at three different temperature, respectively. The adsorption capacity of CMC-FMBON is higher than FMBON from the results, which indicated the addition of CMC enhanced the adsorption capacity. At low initial E2 concentration, the adsorption capacity increased rapidly, which might be due to the fact that there are large number of massive active sites available during the adsorption process. However, the trend became slow as the E2 concentration further increased, indicating that most of the accessible active sites were occupied in the later adsorption process. Similar studies were observed for removing E2 by few-layered graphene oxide [12]. The adsorption capacity of CMC-FMBON were 84.20, 80.21, and 65.23 mg/g under different temperatures (298, 308, and 318 K, respectively). As the temperature increased, the quality adsorbed of E2 gradually decreased. This results indicated that the process of adsorption might be a spontaneous process, which suggested that the low temperature were conducive to the removal of E2.

To investigate the adsorption process, two isotherm models (Langmuir and Freundich) [10,45] were used to fit the experiment results. The equations of two isotherm models are displayed in the following:

Langmuir equation:

(6) $$q_{e}=\frac{K_{L}q_{m}C_{e}}{1+K_{L}C_{e}{}_{}}\;$$

Freundich equation:

(7) $$q_{e}=K_{f}C_{e}^{N}\;$$

where *q*_e_ (mg/g) is the amount of contaminants sorbed, *q*_m_ (mg/g) is the maximum adsorption amount. *C*_e_ (mg/L) is the equilibrium concentration of pollutant in the solution. *K_L_* (L/g) is a constant related to the affinity of the binding sites. *K*_f_ and *N* are the Freundlich constants, which are related to the amount of adsorption and intensity, respectively.

Two adsorption isotherms of E2 on FCMC-FMBON and FMBON are provided in Figure 5. The fitting parameters calculated by the isotherm models are shown in Table 2. Through analysis of the isotherm parameters, it could be clearly found that the Langmuir model could fit the experiment results better than the Freundich model, which indicated that a multilayer heterogeneous coverage of E2 at the interface of the materials was formed.

To further investigate the adsorption properties of E2 onto CMC-FMBON and FMBON, the thermodynamic analysis results was conducted. The thermodynamic parameters (Δ*G*^0^, Δ*H*^0^*,* and Δ*S*^0^) were calculated from the following equations and the calculated results are shown in Table 3.

(8) $$\Delta G^{0}=-RT\ln K^{0}\;$$

(9) $$\ln K^{0}=\frac{\Delta S^{0}}{R}-\frac{\Delta H^{0}}{RT}\;$$

where *R* (8.314 J/mol *K*) is the universal gas constant, *T* (*K*) is the absolute temperature. *K*^0^ is the adsorption equilibrium constant calculated by plotting ln *K*_d_ (*K*_d_ *= q*_e_*/C*_e_) versus *C*_e_ and extrapolating *C*_e_ to zero. Δ*H*^0^ and Δ*S*^0^ were calculated from the slope and intercept of the plot of ln *K*^0^ vs. 1*/T*.

The values of Δ*G*^0^ were found to be negative with the temperature decrease, which suggested that this adsorption process involved a favorable and spontaneous process. The negative value of Δ*H*^0^ indicated that the adsorption of E2 was exothermic process, and thus the adsorption capacity of E2 decrease as the temperature increased. The values of Δ*S*^0^ were also found to be negative, which confirmed that the randomness of adsorbate-adsorbent interface decreased during the adsorption progress.

### 3.3. The External Environmental Factors on Removal Process of E2

#### 3.3.1. Effect of Initial Solution pH on E2 Removal Process

**Figure 6:**
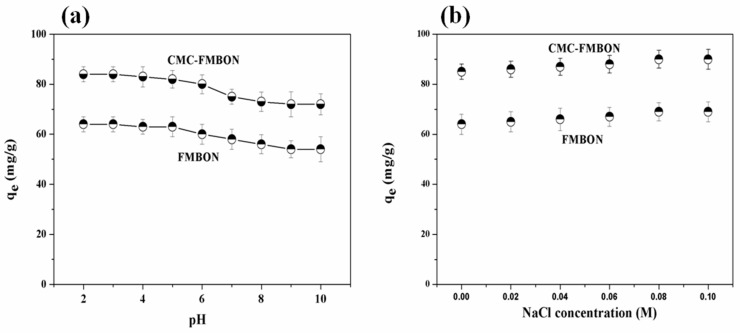
Effect of the (**a**) solution pH and (**b**) ionic concentration on the adsorption of E2 using CMC-FMBON and FMBON.

Solution pH affects both the E2 speciation and zeta potential of particles. Figure 6a showed the influences of initial solution pH on E2 removal by CMC-FMBON and FMBON with pH range from 2.0 to 10.0. It was clearly found that as the pH was increased from 2.0 to 10.0, the adsorption capacity was significantly lowered. These phenomena might be caused by the change of the surface charge of adsorbents and the E2 speciation at different pH. As seen, the zeta potential of CMC-FMBON and FMBON kept negative and reduced along at the whole range of pH. And the negative charge would be improved while increasing initial solution pH. It was reported that the p*K*_a_ of E2 is 10.4 [46]. E2 would begin to deprotonate near pH 9.0. A research study by Sun et al. [33] reported that E2 was easily dissociated in a weakly alkaline solution. The E2 molecules slowly became negative after deprotonating. The negatively charged sites dominated at the higher pH, which enlarged the repulsive electrostatic between the adsorbent and E2. Besides, deprotonation would destroy the hydrogen bonds between adsorbents and E2 by the replacement the protons on E2 molecules. Therefore, the adsorption capacity of CMC-FMBON and FMBON decreased as the pH in the solution increased. It suggested that the removal of E2 by CMC-FMBON was obviously dependent on pH and the maximum adsorption occurring when pH was acidic.

#### 3.3.2. Effect of Ionic Strength and Coexisting Ions on E2 Removal

As known, there are many pollutants including inorganic and organic pollutants and toxicants and salts in wastewater. Some of them may affect the adsorption process. Therefore, the impact of ionic strength on the E2 removal process was investigated through experiment studies constructed by varying the concentration of NaCl solution from 0 to 0.1 M. The experiment results are described in Figure 6b. It could be seen that the adsorption capacity of E2 by CMC-FMBON and FMBON was increased in the presence of NaCl in the solution. There may be two potential effects in E2 adsorption process by CMC-FMBON and FMBON: (1) Adding electrolyte into solution lead to the decreases or increases of other electrolyte or non-electrolyte, which called the salt-out or salt-in effect [47], the salt-in effect reduced the hydrophilic organic compounds and caused their solubility to decrease, which resulted in the contaminants to be more easily adsorbed by hydrophobic adsorbent. (2) The increase of ionic strength weaken the electrostatic repulsion effect, which was favorable to the removal of E2. Therefore, the adsorption capacity of CMC-FMBON and FMBON for E2 improved in the presence of NaCl.

**Figure 7:**
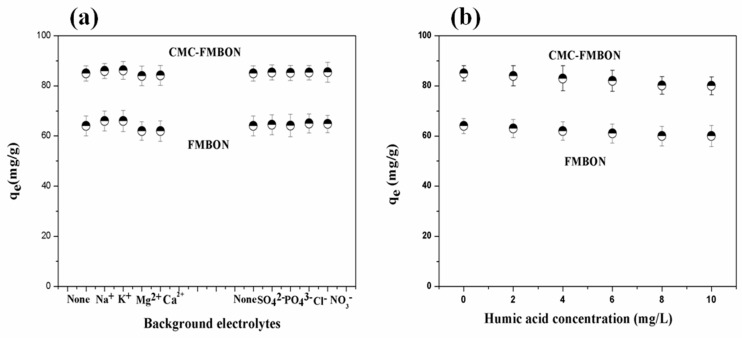
Effect of (**a**) background electrolytes and (**b**) humic concentration on the removal of E2 by CMC-FMBON and FMBON.

The influences of competing ions, including Na^+^, Mg^2+^, K^+^, Ca^2+^, Cl^−^, SO_4_^2^^−^, NO_3_^−^, and PO_4_^3^^−^ on removing E2 by CMC-FMBON and FMBON were investigated, respectively. The corresponding results are illustrated in Figure 7a. For cations, the adsorption capacity in the presence of monovalent cations (Na^+^ and K^+^) was higher than in the presence of divalent cations (Mg^2+^ and Ca^2+^). They penetrate into the diffuse double layer surrounding adsorbents surfaces of ions may result in the removal of repulsive interaction and promote the formation of a more compact aggregation structure, which is referred to as the squeezing-out effect. This observation could be attributed to the squeezing-out effect dominated in the presence of divalent cations, which increased the solubility of E2 in the solution. Figure 7a displays the influence of coexisting anions (Cl^−^, SO_4_^2^^−^, NO_3_^−^, and PO_4_^3^^−^) on the E2 removal onto CMC-FMBON and FMBON. It was found that the adsorption capacity of adsorbent for E2 enhanced in the presence of anions. These observation might be due to the electrostatic repulsion between the negatively charged adsorbents surface and anions. The results suggested that the CMC-FMBON were able to provide some active sites in the presence of coexisting ions.

#### 3.3.3. Effect of Humic Acid on E2 Removal

Humic substances are widely found in natural water, which are mainly derived from the decomposition of natural organic compounds. The impact of humic acid on the E2 removal are exhibited in Figure 7b. The adsorption capacity decreased as the humic acid concentration was increased in water. It had been reported that humic acid contained a lot of functional groups such as carboxylates and phenolic hydroxyls [48]. The dissociation of functional groups in water resulted in the formation of negative charges bound that can interact with organic containments in water. And the formation of soluble E2-humic acid complexes in water [49], which might be attributed to decrease of E2 adsorption. There are other reasons that might explain the results that the interaction of humic acid and adsorbents can occupy some of the surface adsorption sites of adsorbents thereby reducing the adsorption capacity of E2 [49].

#### 3.3.4. Comparisons with the Other Materials

Table 4 compared the maximum adsorption capacity of other adsorbents for E2. As seen, the removal capacity of E2 by the CMC-FMBON was relatively higher than other adsorbents, which suggested that the CMC-FMBON might be a higher efficiency adsorbent for the removal E2 in aqueous system.

#### 3.3.5. Adsorption Mechanism of CMC-FMBON for E2

To study the molecular interaction mechanism of CMC-FMBON with E2, Figure 3c compared the FTIR spectra of CMC-FMBON before and after E2 adsorption. Various iron oxides were existed in the previous studies: 560 and 580 cm^−1^ for ferrihydrite and magnetite, respectively. However, the results of FTIR spectra did not display these peaks, which indicated that the presence of Mn could have decreased the band strength of Fe–O groups. Two peaks at 1115 and 1057 cm^−1^ were ascribed to the bending vibration of the iron hydroxides (Fe–OH) hydroxyl groups [52]. The peak at 1115 and 1057 cm^−1^ disappeared and could not be detectable after adsorption of E2. This observation might be that the E2 species replaced the hydroxyl groups on the surface of CMC-FMBON. Compared to CMC-FMBON, it can be observed that there are quite a few peaks in the FTIR spectrum of CMC-FMBON after E2 adsorption. These peaks of 500–1200 cm^−1^ were attributed to the peaks of E2 molecules, which suggested that E2 molecules had been adsorbed onto the CMC-FMBON surfaces. The peak at 1625 cm^−1^ can be assigned to the stretching vibration of aromatic C=C bonds. The peaks of C=C bonds shifted from 1625 cm^−1^ to 1638 cm^−1^ after adsorption E2, which indicated that π-π interactions existed between CMC-FMBON and E2. The peak at 1061 cm^−1^ belonged to C-O bonds. A shift was observed from 1061 to 1085 cm^−1^. These shifts could be attributed to the increased intensity of hydrogen bonds between E2 and CMC-FMBON. In addition, the representative chromatogram before and after E2 adsorption are exhibited in Figure S2. As can be seen, the higher peak (retention time = 15 min) was assigned to the E2 peak, which was detected during the initial reaction. The E2 peak decreased after reaction and two new peaks (retention time = 21.5 and 24 min) appeared, which indicated that E2 might be oxidatively degraded by CMC-FMBON. That results are consistent with the observation of the FTIR analysis results. In our study, the changes of these peaks suggest that π-π interactions, hydrogen bonds, and oxidation are adsorption mechanisms of E2 onto CMC-FMBON.

#### 3.3.6. Desorption and Regeneration Analysis

**Figure 8:**
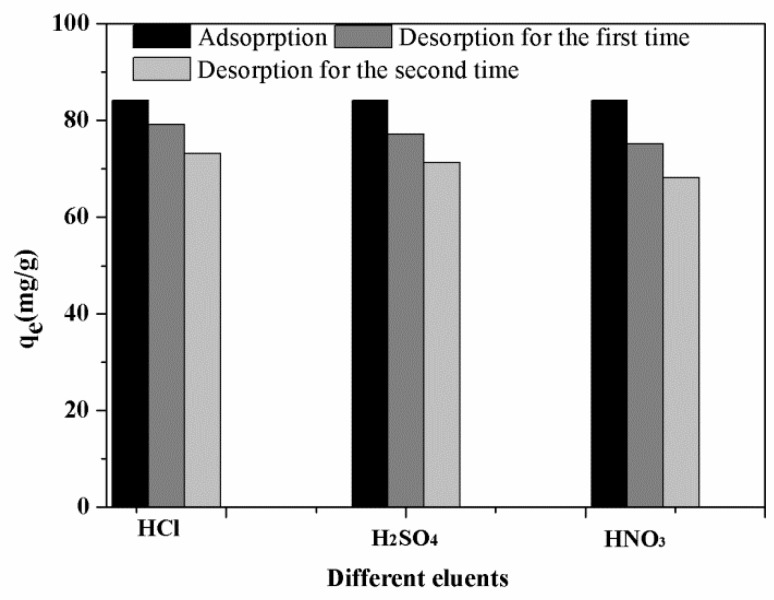
Desorption efficiency of CMC-FMBON for E2 using different eluants.

To investigate the reusability and application prospective of CMC-FMBON for the E2 removal from aqueous system, the desorption/regeneration cycle experiment was explored by using different eluants (0.1 M HCl, H_2_SO_4_, and HNO_3_). The experimental results are displayed in Figure 8. Compared to the different eluants, the desorption of CMC-FMBON for E2 was found better in the presence of 0.1 M HCl, which might be explained by the smaller size Cl^−^ ions than NO_3_^−^ or SO_4_^2−^. It was observed the adsorption capacity was 84.20 mg/g in the first adsorption, after two desorption cycles by 0.1 M HCl, the adsorption ability of E2 onto CMC-FMBON still remained a high adsorption capacity. The excellent regeneration property of CMC-FMBON suggested that it could be economical, and a potential adsorbent for E2 removal from water.

## 4. Conclusions

This study synthesized Fe–Mn binary oxides nanoparticles by using CMC as a stabilizer and investigated adsorption capacity for E2 under external environmental factors. The adsorption capacity of CMC-FMBON for E2 was significantly higher than that of FMBON. The maximum removal efficiency of CMC-FMBON and FMBON for E2 were 124.10 and 98.14 mg/g at 298 K, respectively. The kinetics and isotherms could be interpreted by pseudo-second-order and Langmuir model, respectively. The adsorption capacity was affected by the initial solution pH and the low pH was favorable for E2 adsorption. The E2 adsorption was enhanced by the increase of ionic strength in water. The adsorption capacity in the presence of monovalent cations (Na^+^ and K^+^) was higher than in the presence of divalent cations (Mg^2+^ and Ca^2+^). The adsorption capacity in the presence of background electrolyte anions was increased slightly and decreased with increasing in humic acid concentration. The high adsorption capacity of CMC-FMBON might be oxidation, hydrogen bonds, and π-π interactions. The high removal capability, green and cost-effective CMC-FMBON makes it advantageous and promising for wastewater treatment.

## Figures and Tables

**Table 1:** The kinetics parameters for E2 adsorption by CMC-FMBON and FMBON.

Adsorbent	*q*_e. exp_ (mg/g)	Pseudo-First-Order			Pseudo-Second-Order		
		*R* ^2^	*q*_e_ (mg/g)	*K*_1_ (g/mg·min)	*R* ^2^	*q*_e_ (mg/g)	*K*_2_ (g/mg·min)
CMC-FMBON	84.20	0.96	80.47	0.03	0.98	82.23	0.04
FMBON	60.32	0.96	63.28	0.04	0.98	55.21	0.41

**Table 2:** The isotherm parameters for E2 removal by CMC-FMBON and FMBOBN.

Adsorbent	T	Langmuir Isotherm			Freundich Isotherm			
		*K_L_*	*q*_e_	*R* ^2^	*K_F_*	*q*_e_	*n*	*R* ^2^
CMC-FMBON	298 K	0.20	124.10	0.98	55.58	142.30	1.11	0.93
	318 K	0.14	112.22	0.98	29.32	125.02	1.23	0.93
	338 K	0.10	100.52	0.98	20.60	98.01	1.10	0.95
FMBON	298 K	0.39	98.14	0.97	50.12	95.21	0.96	0.91
	318 K	0.12	84.41	0.99	25.33	81.24	0.95	0.96
	338 K	0.08	75.21	0.98	15.48	78.32	1.02	0.94

**Table 3:** Thermodynamic parameters for E2 removal onto CMC-FMBON and FMBON.

Adsorbents	Temperature (*T*)	ln *k*_0_	Δ*G*^0^ (kJ/mol)	Δ*H*^0^ (kJ/mol)	Δ*S*^0^ (J/K·mol)	*R* ^2^
CMC-FMBON	298 K	1.21	−2.24	−8.846	−12.27	0.96
	318 K	1.04	−1.91			
	338 K	1.12	−1.82			
FMBON	298 K	0.811	−2.15	−7.65	−20.12	0.97
	318 K	0.696	−1.74			
	338 K	0.511	−1.12			

**Table 4:** Comparison of removal capacity of E2 via various materials.

Adsorbent	Adsorption Capacity (*q*_e_ (mg/g))	References
Few-layered graphene oxide nanosheets	149.9	[12]
Sing-walled carbon nanotubes	27.2	[50]
Activated carbons	21.3–67.6	[2]
Magnetic graphene oxide	85.80	[13]
hydrochar-FMBO	49.77	[3]
ACM (activated carbon purchased from Merck)	5.07	[51]
CS (coffee waste and sawdust)	4.95	[51]
CMC-FMBON	124.10	This study

## Supplementary Materials

The following are available online at http://www.mdpi.com/1660-4601/15/10/2218/s1, Figure S1: EDX spectra of (a) FMBON (b) CMC-FMBON (c) E2 adsorbed FMBON (d) E2 adsorbed CMC-FMBON, Figure S2: The representative chromatogram before (a) and after (b) reaction of 15 h.
