# Supplementary material for: Fabrication of Stabilized Fe–Mn Binary Oxide Nanoparticles: Effective Adsorption of 17β-Estradiol and Influencing Factors
Source: Int J Environ Res Public Health. 2018 Oct 11;15(10):2218. doi: 10.3390/ijerph15102218 (PMC6210389; doi:10.3390/ijerph15102218)
Supplement: Supplementary file 1 [file ijerph-15-02218-s001.pdf]

International Journal of
Environmental Research and Public Health

MDPI

Supplementary Material

Fabrication of Stabilized Fe–Mn Binary Oxide Nanoparticles: Effective Adsorption of 17β-Estradiol and Influencing Factors

Qimeng Ning [1,2,3], Zhihong Yin [1,2,*], Yunguo Liu [1,2], Xiaofei Tan [1,2], Guangming Zeng [1,2], Luhua Jiang [1,2], Shaobo Liu [4,5], Sirong Tian [1,2], Ni Liu [1,2] and Xiaohua Wang [1,2,3]

1 College of Environmental Science and Engineering, Hunan University, Changsha 410082, China; ningqimeng@163.com (Q.N.); hnuliuyunguo@gmail.com (Y.L.); Tanxf@hnu.edu.cn (X.T.); zgming@hnu.edu.cn (G.Z.); Jiangluhua@hnu.edu.cn (L.J.); Tsirong@hnu.edu.cn (S.T.); meet_liuni@hnu.edu.cn (N.L.); Wangxiaohua6999@126.com (X.W.)
2 Key Laboratory of Environmental Biology and Pollution Control (Hunan University), Ministry of Education, Changsha 410082, China
3 School of architecture and urban planning. Hunan City University. Yiyang 413000, China
4 School of Architecture and Art, Central South University, Changsha 410082, China; Liushaobo23@163.com
5 School of Metallurgy and Environment, Central South University, Changsha 410083, China
* Correspondence: Yinzhihong@hnu.edu.cn; Tel.: +86-731-8864-9208

17β-estradiol detection:

The E2 concentration was determined using a fluorescence quenching method via an F-4500 fluorescence spectrophotometer (Hitachi, Japan). A xenon lamp (450 W) was chosen as the excitation source, and the slits of excitation (Ex) and emission (Em) were remained at 5 nm. The fluorescence intensity of E2 was set to Ex/Em=280 nm/310 nm [1]. The calibration curve was measured by using E2 dissolved in truly dissolved solution at concentrations of 0.05-8.0 mg/L. Meanwhile, the background fluorescence intensities of 0.01 M NaCl solution were measured and subtracted under the same conditions.

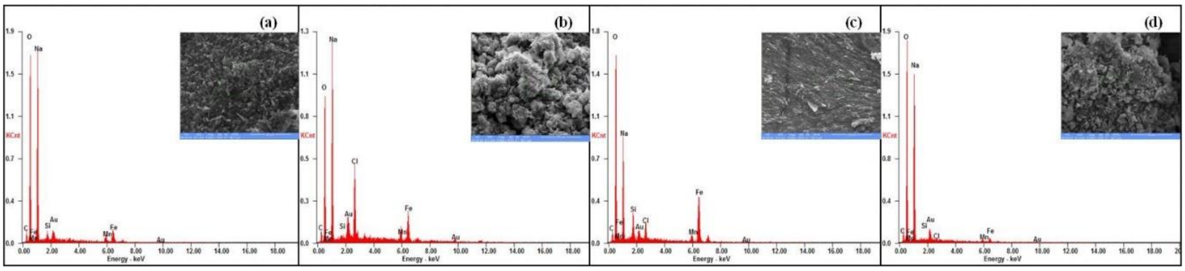

Figure S1. EDX spectra of (**a**) FMBON (**b**) CMC-FMBON (**c**) E2 adsorbed FMBON (**d**) E2 adsorbed CMC-FMBON.

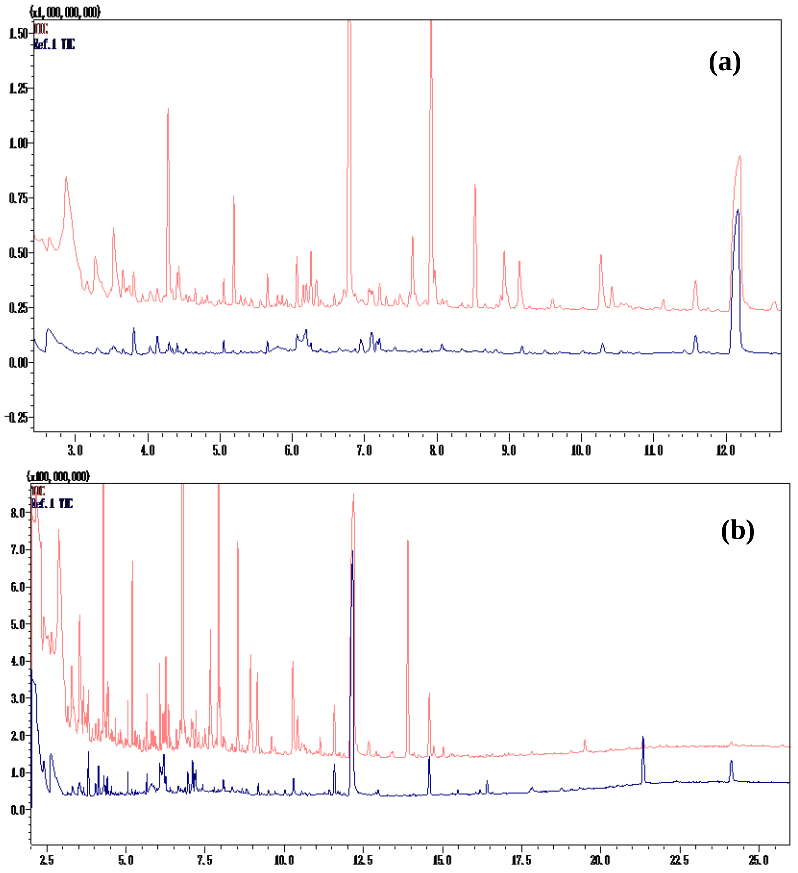

Figure S2. The representative chromatogram before (**a**) and after (**b**) reaction of 15 h.

[1] L.H. Jiang, Y.G. Liu, G.M. Zeng, F.Y. Xiao, X.J. Hu, X. Hu, H. Wang, T.T. Li, L. Zhou, X.F. Tan, Removal of 17β-estradiol by few-layered graphene oxide nanosheets from aqueous solutions: External influence and adsorption mechanism, *Chem. Eng. J.* **2016**,*284*,93-102.

© 2018 by the authors. Submitted for possible open access publication under the terms and conditions of the Creative Commons Attribution (CC BY) license (http://creativecommons.org/licenses/by/4.0/).
